# The Impact of Non-Coding RNA on Inflammation and Airway Remodeling in Asthma Related to Obesity: State-of-the-Art and Therapeutic Perspectives

**DOI:** 10.3390/jcm14207161

**Published:** 2025-10-11

**Authors:** Maria Kachel, Wojciech Langwiński, Aleksandra Szczepankiewicz

**Affiliations:** 1 Molecular and Cell Biology Unit, Department of Pediatric Pulmonology, Allergy and Clinical Immunology, Poznan University of Medical Sciences, Szpitalna 27/33, 60-572 Poznań, Poland; 2 Experimental Medicine Centre, Poznan University of Medical Sciences, Rokietnicka 8, 60-806 Poznań, Poland

**Keywords:** microRNA, long non-coding RNA, circular RNA, asthma, obesity

## Abstract

Asthma is a chronic respiratory disease affecting over 262 million people worldwide, with obesity-associated asthma emerging as a distinct endotype of increasing prevalence characterized by metabolic inflammation and airway remodeling. Unlike allergic asthma, this phenotype is driven by chronic low-grade inflammation, originating from hypertrophic and hypoxic adipose tissue. This dysregulated state leads to the activation of pro-inflammatory pathways and the secretion of cytokines, contributing to airway dysfunction and remodeling. Recent evidence highlights non-coding RNAs (ncRNAs) as key regulators of these processes. MicroRNAs (miRNAs), long non-coding RNAs (lncRNAs), and circular RNAs (circRNAs) influence inflammation and remodeling by modulating immune cell polarization, cytokine secretion, extracellular matrix composition, and airway smooth muscle cell (ASMC) proliferation. Notably, H19, MEG3, GAS5, miR-26a-1-3p, and miR-376a-3p have been implicated in both asthma and obesity, suggesting their role in linking metabolic dysfunction with airway pathology. Moreover, ncRNAs regulate Treg/Th17 balance, fibroblast activation, and autophagy-related pathways, further influencing airway remodeling. Our in silico analysis highlighted the IGF1R signaling pathway as a key enriched mechanism, linking selected ncRNAs with metabolic dysregulation and inflammation in obesity-related asthma. This paper reviews how ncRNAs regulate inflammation and airway remodeling in obesity-associated asthma, emphasizing their potential molecular links between metabolic dysfunction and airway pathology.

## 1. Introduction

Asthma is a chronic respiratory disease that, according to the World Health Organization (WHO) in 2019, affected 262 million people and caused 455,000 deaths [1]. This means that in 2019, there were 478 new patients diagnosed with asthma for every 100,000 people. In contrast, the 2021 figures show that the incidence of asthma increased to 3340 cases for every 100,000 people [2]. The disease affects all age groups; however, it is most common among children and teenagers (10.4%) [3]. Based on a meta-analysis by Yuan et al., approximately 22 million children develop asthma each year, especially in preschool age (0–4 years) [2].

Asthma symptoms include persistent cough, wheezing, shortness of breath, chest tightness, and dyspnea. Additionally, patients may experience asthma attacks in response to triggers such as inhaled allergens (house dust mite, pollen, animal fur, and molds), viral infections, smoke, and air pollution. Clinical diagnosis is determined by identifying symptoms and confirming documented variations in expiratory airflow limitation through a pulmonary function test [1,4]. Current understanding of the disease focuses on the diversity of phenotypes and endotypes caused by different biological mechanisms and thus greatly influences treatment strategies and outcomes.

Asthma symptoms result from an inflammatory response to environmental triggers and airway remodeling caused by immune system activation [5,6]. As a result, asthma phenotypes classification is based on the type of inflammation involved—Th2-high and Th2-low. Type 2–high asthma demonstrates elevated levels of eosinophils and T2 cytokines such as IL-4, IL-5, and IL-13. In contrast, type 2–low asthma involves neutrophilic inflammation, although its exact mechanisms are yet to be fully understood [5].

In recent years, the rising prevalence of obesity—affecting 41.9% of the U.S. population between 2017 and 2020 and 12.5% of the population worldwide in 2022—has been accompanied by an increase in cases of obesity-related asthma [7,8]. This comorbidity results mainly from mechanical stress on the airways, metabolic dysregulation, and low-grade chronic systemic inflammation [9]. Obesity can contribute to airway dysfunction and the development of asthma symptoms [10]. Complex clinical symptoms and difficulties in treatment have shifted focus to a better understanding of the regulatory mechanisms and pathways underlying this asthma endotype, particularly in the aspect of poor response to current antiasthmatic therapies. Non-coding RNAs are the most promising players that could serve as potential biomarkers allowing for the differentiation of asthma endotypes.

Non-coding RNAs (ncRNAs) are RNA molecules that do not encode proteins. Although recent studies suggest that some of them might contain open reading frames (ORFs), and therefore may have coding potential [11]. NcRNAs are key regulators of gene expression, including transcription, RNA processing, translation, epigenetic modifications, and cellular signaling, thereby controlling many cellular processes. It is a diverse group of molecules that includes short ncRNAs, mainly microRNAs (miRNAs), long non-coding RNAs (lncRNAs), circular RNAs (circRNAs), and many others [12].

In this review, we aimed to summarize the current knowledge on the role of non-coding RNAs in the pathomechanisms linking obesity and asthma. Since this endotype is not fully understood yet, we decided to take a different approach and also showcase current knowledge regarding the pathophysiology of asthma and obesity in order to highlight gaps, where further research is needed to better understand these processes and improve diagnostic and therapeutic approaches. Moreover, we also performed in silico analysis, including selected ncRNAs, to further feature their potential functional significance in the context of obesity-related asthma. This approach allowed us to supplement the current literature with conclusions from computational analysis to indicate the most promising regulators in asthma related to obesity.

## 2. Classes of Non-Coding RNAs

There are multiple classes of non-coding RNAs (ncRNAs), which can be classified based on their genomic localization or transcript length, including small nuclear RNAs (snRNAs), small nucleolar RNAs (snoRNAs), PIWI-interacting RNAs (piRNAs), transfer RNAs (tRNAs), and ribosomal RNAs (rRNAs), among others. While these groups are indispensable for fundamental processes such as splicing or translation, their direct involvement in the pathophysiology of obesity-associated asthma has not yet been demonstrated. Therefore, this review focuses on the three groups of ncRNAs that have been most extensively studied in this context—microRNAs, long non-coding RNAs, and circular RNAs.

Based on the available literature, the most extensively studied ncRNAs are microRNAs (miRNAs). These small endogenous RNAs, typically 19–25 nucleotides long, participate in the regulation of gene expression. After their processing and incorporation into the RNA-induced silencing complex (RISC), the mature miRNA binds to target mRNAs via the seed region, a 2–8 nucleotide sequence at the 5′ end of the microRNA, leading to translational repression or mRNA degradation depending on the degree of complementarity [13,14]. MicroRNA activity regulates the expression of up to 60% of human protein-coding genes, and can be observed not only in cytoplasm, but also in extracellular vesicles, a promising target for clinical trials exploring microRNA-based therapies [13,15,16].

Another class of ncRNAs includes long non-coding RNAs (lncRNAs), molecules at least 200 nucleotides long that may contain a non-functional “pseudo-open” reading frame [17]. Roughly 50% of lncRNAs have poly-A tails, and almost 98% of them undergo splicing, which makes them resemble mRNAs. LncRNAs can be grouped based on their genomic localization and structure. LncRNAs participate in chromatin remodeling, regulation of transcription, splicing, and translation. They also contribute to the formation of subcellular structures and act as miRNA sponges, thus affecting their ability to target mRNAs. Moreover, lncRNAs may influence signaling pathways in a cis- and trans-acting manner, modulating neighboring and distal processes [17,18].

The other emerging group of ncRNAs is circRNAs, characterized by a circular, covalently closed RNA structure. This unique feature means that they lack 5′ and 3′ ends, which, in comparison to miRNAs and lncRNAs, significantly increases their stability and resistance to nucleases. CircRNAs are formed in a process called back-splicing, which utilizes the canonical spliceosome machinery. Recent studies suggest that long intronic sequences and repetitive Alu elements may play a crucial role in their formation, facilitating back-splicing by competing with linear splicing of pre-mRNA. They are present both in the cytoplasm and the nucleus, and their intracellular distribution depends on their size, potentially influencing their functional roles. CircRNAs mainly act as microRNA sponges—via binding and sequestering miRNAs—they regulate their activity, thus controlling transcription and translation [19].

## 3. Inflammation

The main pathophysiological mechanism underlying obesity-related asthma is chronic systemic low-grade inflammation. In obese patients, this process is also described as metabolic inflammation or meta-inflammation, which differs from classical allergic inflammation observed in non-obese asthmatics [20,21]. Meta-inflammation originates from an excess of dysfunctional adipose tissue, which in lean individuals secretes a balanced combination of pro- and anti-inflammatory cytokines. However, with the progression of obesity, adipose tissue undergoes hypertrophy, leading to increased distances between adipocytes and capillaries, resulting in adipocyte hypoxia and subsequent apoptosis. As a result, key pro-inflammatory cytokines such as TNFα, IL-1β, IL-6, and IL-18 are secreted following caspase-1 activation triggered by the NLR family pyrin domain containing 3 (NLRP3) inflammasome, thus initiating a sustained pro-inflammatory cascade [21,22].

Recent findings have highlighted the involvement of non-coding RNAs in modulating obesity-related inflammation and asthma pathology. Experiments in animal models of asthma have shown significant downregulation of miR-182-5p, and further in vitro studies indicated its role in the inhibition of the NLRP3/IL-1β pathway and suggested that dysregulation of this miRNA leads to an excessive inflammatory response, thus worsening airway pathology [23]. Among lncRNAs, LINK-A promoted adipose hypertrophy, insulin resistance, and systemic inflammation by enhancing IL-6 and IL-1β secretion through activation of the LINK-A/HB-EGF/HIF1α loop [24]. In this context, another lncRNA, GAS5, has been identified as a potential biomarker of asthma, showing higher expression in asthmatic patients compared to healthy controls. That effectively differentiates between individuals with obesity-related asthma and those with obesity alone. While its precise mechanism is not fully understood, GAS5 has been implicated in various inflammatory processes, apoptosis regulation, and cell survival [22].

At the cellular level, inflammation involves increased infiltration of macrophages, with a shift towards the pro-inflammatory M1 phenotype. The proportion of M1 macrophages in adipose tissue increases from 4% up to 12% as obesity progresses, leading to the release of inflammatory cytokines such as IL-1β, TNFα, and IL-6, that sustain a chronic inflammatory state [25]. This phenomenon extends beyond adipose tissue, contributing to systemic immune activation and increased risk of metabolic diseases such as insulin resistance and type 2 diabetes [20]. Importantly, ncRNAs have been implicated in regulating the inflammatory environment. Pan et al. demonstrated that miR-34a is secreted in exosomes from mature adipocytes and transported into adipose-resident macrophages, thus suppressing M2 polarization by inhibiting Krüppel-like factor (Klf4), and shifting macrophages towards a pro-inflammatory phenotype associated with metabolic inflammation [24].

Among the ncRNAs linked to both obesity and asthma, miR-26a-1-3p and miR-376a-3p have been identified as potential regulators of insulin-like growth factor (IGF) signaling, which plays a role in metabolic inflammation. Studies investigating the targets of these miRNAs revealed differential expression of IGFBP-3 between asthma patients and healthy individuals, further supporting the involvement of IGF signaling in disease pathogenesis. Interestingly, in obese asthma patients, elevated levels of miR-26a-1-3p and IGF-1R were observed, while miR-376a-3p and IGFBP-3 levels were decreased. Moreover, miR-26a-1-3p showed a direct correlation with body mass index (BMI), whereas IGFBP-3 exhibited an inverse correlation, suggesting a potential role for these miRNAs in bridging metabolic dysfunction and airway pathology [26].

Adipose tissue acts as an endocrine organ, secreting adipokines that play a crucial role in regulating inflammation and immune responses. Among them, leptin and adiponectin gained the most attention due to their opposing effects on inflammation and metabolic homeostasis. Leptin, which regulates appetite and metabolism, was significantly increased in obese individuals [20,21,27], and its excess has been linked to systemic inflammation, insulin resistance, and airway hyperresponsiveness. Leptin also stimulates the production of pro-inflammatory cytokines such as IL-17, TNFα, and IL-6, intensifying inflammation in both adipose tissues and the lungs. Previous studies suggested that airway epithelial cells express leptin receptors, allowing leptin to exert a direct impact on airway reactivity. Sideleva et al. performed methacholine challenge tests in female patients (obese without asthma vs. asthmatic and obese) and found that the higher the leptin expression in visceral fat, the lower the bronchial reactivity. Interestingly, leptin expression showed a higher correlation to methacholine response than BMI alone. This observation is supported by findings showing that leptin levels in visceral fat correlated with increased airway resistance and inflammation, providing a mechanistic link between obesity and asthma [20,21]. Additionally, elevated leptin levels enhanced the activation of Th17 cells, contributing to the neutrophilic inflammation specific for non-eosinophilic obesity-related asthma [20]. Furthermore, lncRNA-MEG3 has been associated with the regulation of Treg/Th17 balance via modulation of RORγt and Foxp3. Research by Qiu et al. demonstrated that MEG3 acts as a competing endogenous RNA (ceRNA) by silencing miR-17. Silencing the expression of the aforementioned ceRNA in an in vitro study on a CD4 + T cell line resulted in increased IL-17 expression, enhancing Th17 differentiation. Thus, reduced expression of lnc-MEG3 could influence the development of Th2-low asthma symptoms [28].

Conversely, adiponectin—an adipokine with anti-inflammatory properties—is significantly reduced in obesity, which further provokes inflammation and metabolic dysfunction [20,21]. In lean individuals, adiponectin promotes M2 macrophage polarization, reversing the inflammatory effects of leptin and suppressing the production of TNFα and IL-6. However, its deficiency in obesity is associated with more severe airway inflammation and enhanced remodeling, contributing to the enhanced asthma symptoms observed in obese patients [20]. Interestingly, some studies suggested that adiponectin may exhibit pro-inflammatory effects in specific pathological contexts, such as rheumatoid arthritis and inflammatory bowel disease, highlighting the complexity of its function [20]. The regulation of adipokine-mediated inflammation is further influenced by ncRNAs. Lischka et al. reported that miR-192, upregulated in obesity, correlated with increased levels of inflammatory markers such as TNFα and IL-1Ra, but decreased adiponectin levels [29]. Additionally, Han et al. demonstrated that lncRNA SNHG14 is implicated in adipose inflammation by interacting with miR-497a-5p, which in turn regulates BACE1 expression. BACE1 is a protein involved in the regulation of energy homeostasis and metabolism, promoting ER stress and the development of inflammation. Knockdown of SNHG14 decreased IL-1β, IL-6, and TNFα secretion, therefore decreasing adipocyte inflammation and endoplasmic stress [30]. A study by Wang et al. demonstrated that circTXNRD1, which is upregulated in asthma, serves as a molecular sponge for miR-892a. This interaction leads to increased COX-2 expression, promoting IL-6 secretion and aggravating airway inflammation. Knockdown of circTXNRD1 significantly reduced IL-6 levels, indicating its potential role in modulating airway immune responses [31]. Moreover, Zhang et al. found that lncRNA-AK149641 downregulation in the OVA-induced asthma mouse model inhibited airway inflammation by reducing TNFα and IL-6 secretion, thus improving airway resistance and compliance during the methacholine challenge test. Moreover, diminishing inflammatory cell infiltration was also observed, possibly through modulation of the NF-κB pathway [32]. A comprehensive summary of key ncRNAs involved in inflammation is provided in Table 1.

## 4. Airway Remodeling

Airway remodeling is recognized as an important pathological mechanism in asthma that includes structural changes in the bronchial wall [53]. This process involves repetitive epithelial damage [53,54], extracellular matrix (ECM) reorganization [53,55], hypertrophy and hyperplasia of airway smooth muscle cells (ASMCs) [53]. Moreover, due to dynamic changes within the lung tissue, increased angiogenesis was also observed, which further facilitates the recruitment of more inflammatory cells, enhancing the remodeling process [56,57]. Interestingly, recent studies reported no significant differences in airway remodeling mechanisms between eosinophilic and neutrophilic inflammation, suggesting a common pathological pathway underlying this process [53,58,59]. Although obesity may not directly induce airway remodeling, it is increasingly recognized as an important risk factor that exacerbates this process. Metabolic disorders associated with excess adipose tissue, including insulin resistance, dysregulated adipokine secretion, may enhance airway hyperresponsiveness, leading to structural changes. Moreover, the mechanical effects of obesity, particularly the accumulation of fat in the abdominal and thoracic regions, lead to reduced lung volumes and increased airway resistance, which further promote remodeling of the airways [60,61,62].

Following injury, the airway epithelium releases growth factors such as epidermal growth factor (EGF) and transforming growth factor beta (TGF-β) to initiate epithelial repair through epithelial-to-mesenchymal transition (EMT) [57,63]. Yang et al. [64] found that downregulation of miR-448-5p in asthmatic mice correlated with increased Six1 expression, a gene responsible for proliferation and tissue development, which led to TGF-β1-mediated EMT and fibrosis. Moreover, Wang et al. [65] discovered that miR-451a-5p was downregulated in the lungs of asthmatic mice. They discovered that miR-451a-5p inhibits the expression of alpha-smooth muscle actin (α-SMA) and CDH11, proteins responsible for maintaining cell integrity and structure. During EMT, interactions within the epithelial-mesenchymal-trophic unit (EMTU) regulate the repair processes, including fibroblast differentiation into myofibroblasts, epithelial cell division, and matrix remodeling, while also regulating immune responses to environmental stimulants [57]. For instance, decreasing the expression of miR-145 increased the expression of epidermal growth factor receptor (EGFR), which regulates mucin 5AC (MUC5AC), and its overexpression in asthma was linked to pathological mucus hypersecretion.

In asthmatic patients, ASMC hypertrophy and hyperplasia contribute to disease severity by increasing airway resistance and decreasing elasticity, which enhances contractility and impairs lung function [57,66]. According to the literature, ASM proliferation is a highly complex process regulated by multiple signaling pathways, including the suppression of apoptotic and anti-proliferative mechanisms, along with upregulation of proliferative signaling cascades [57,67]. Interestingly, previous studies showed that lncRNA MALAT1 promotes the proliferation and migration of ASMcs in asthma by sponging microRNA-216a, thus disrupting the balance of cellular growth and survival pathways [68]. The key pathways involved in ASMC proliferation are PI3K/Akt, p38 MAPK, p42/44 MAPK, TGF-β, and NF-κB, and all are activated in response to airway inflammation and growth factor stimulation. A study performed by Huang et al. [69] showed that lncRNA TUG1 contributes to airway remodeling in asthmatic mice by interfering with miR-181b, leading to HMGB1 overexpression and further activation of the NF-kB pathway. Additionally, pro-inflammatory cytokines such as TNF-α, TGF-β, IL-4, IL-13, and Th17 contribute to ASM hyperplasia by promoting inflammation-driven ASMC growth [57]. Shi et al. [70] found that transfection of miR-130a-3p mimics or miR-142-5p antisense oligonucleotide (ASO) effectively inhibited interleukin-4-induced M2 cytokine production in isolated pulmonary macrophages, further highlighting the role of ncRNAs in inflammatory signaling.

One of the most potent stimulators of airway remodeling upregulated in asthma is platelet-derived growth factor-BB (PDGF-BB), a growth factor that plays a critical role in ASMC proliferation, migration, and ECM synthesis. PDGF-BB activates downstream pathways, including PI3K/Akt, Wnt/βM β-catenin, and NF-kB, that collectively enhance airway remodeling [57]. Emerging studies indicate that circular and long non-coding RNAs, acting as miRNA sponges, disrupt the normal ASM proliferation and migration in airway remodeling, mostly due to pathological pathways activated by PDGF-BB. According to Lin et al. [71], circHIPK3, which acts as a sponge for miR-326, was upregulated in PDGF-stimulated ASMCs. This interaction suppressed miRNA activity, leading to increased expression of stromal interaction molecule 1 (STIM1), a key regulator of calcium signaling and ASMC proliferation. Similarly, an in vitro study revealed that circ_0002594 sponges miR-139-5p, which prevents the downregulation of TRIM8. High expression of this protein promotes the proliferation of AMSCs and Th2 cells, activates NF-kB and NLRP3 pathways, resulting in the progression of airway remodeling and inflammation. Silencing this circRNA was shown to reduce ASMC damage in response to PDGF-BB stimulation [71]. Moreover, circERBB2 has also been shown to drive BDGF-BB-induced ASMC hyperplasia by sponging miR-98-5p, which in turn promotes IGF1R expression, a mediator of ASMC survival and proliferation [72]. On the other hand, lncRNAs such as ANRIL and PVT1 have also been implicated in airway remodeling by modulating key gene expression pathways. ANRIL sequesters miR-7-5p, leading to upregulation of ERG3, a transcriptional factor promoting ASMC proliferation and migration [73]. Similarly, PVT1 has been linked to IL-6 regulation in ASMCs, suggesting its role in inflammation-driven remodeling. Interestingly, studies by Austin et al. [74] show that PVT1 exhibits an opposite regulatory pattern depending on asthma severity, increased in severe asthma not responding to corticosteroid and decreased in corticosteroid-insensitive non-severe asthma, which further suggests its role in steroid resistance mechanisms. A summary of ncRNAs involved in airway remodeling in asthma was presented in Table 2.

## 5. Identifying the Pathways of Selected ncRNA—In Silico Analysis

In the previous paragraphs, we discussed the impact of ncRNAs on inflammation and airway remodeling, along with their potential role in the progression of obesity-related asthma. However, due to the limited availability of the literature addressing this asthma endotype, most of the ncRNAs mentioned above were linked to either asthma or obesity rather than their comorbidity. Nevertheless, the occurrence of certain ncRNAs in both conditions suggests their potential involvement in the pathomechanisms of obesity-related asthma. This highlights a promising direction for future research, emphasizing the need to further explore their functional relevance in this complex disease.

Among the ncRNAs implicated in both asthma and obesity, lncRNAs H19 and MEG3 have emerged as regulators of inflammatory processes, suggesting their potential role in the pathogenesis of obesity-related asthma [28,37,49]. H19 has been identified in both diseases and is involved in the NF-kB signaling pathway. Network analyses of H19 performed by Chen et al. [37] revealed its interaction with multiple miRNAs and a broad range of target genes, showing its extensive regulatory influence in immune responses. Similarly, MEG3 has been shown to influence the Treg/Th17 balance by modulating RORγt and Foxp3 expression, promoting Th17 cell differentiation and shifting immune responses toward a pro-inflammatory phenotype. Mechanistically, MEG3 acts as a ceRNA by sponging miR-17, thereby increasing RORγt expression and enhancing Th17-driven inflammation [28]. In obesity, MEG3 expression correlates with key metabolic markers, including FAS and PPARγ, further implicating it in adipose tissue function and systemic inflammation [49].

Apart from H19 and MEG3, other ncRNAs implicated in both airway remodeling and inflammation across asthma and obesity include TUG1 and miR-192-5p. TUG1 is an lncRNA associated with airway remodeling in asthma, via promoting NF-kB activation [69]. In contrast, in obesity, TUG1 plays an anti-inflammatory role, improving the metabolic function of adipose tissue by downregulating miR-204 and enhancing the GLUT4/PPARγ/AKT pathway [45]. MiR-192 has also been linked to both inflammation and airway remodeling, with opposite effects in obesity and asthma. In obesity, elevated miR-192 has been correlated with pro-inflammatory markers such as IL-1Ra, TNFα, and procalcitonin, and with metabolic dysfunction, dyslipidemia, and nonalcoholic fatty liver disease (NAFLD) [29]. Conversely, in asthma, miR-192-5p downregulation has been linked to increased airway remodeling. Experimental studies showed that increasing miR-192-5p levels suppresses ASMC proliferation and reduces key remodeling markers, including MMP-2, MMP-9, and FGF-23, with simultaneous attenuation of airway inflammation by lowering OVA-specific IgE, IL-4, IL-5, and IL-13 in an animal model. Additionally, miR-192-5p plays a role in autophagy by decreasing ATG7 and beclin-1, while increasing p62, further contributing to the reduction in airway structural changes [76].

To further highlight the functional significance of selected ncRNAs in the context of obesity-related asthma, we performed a preliminary in silico analysis based on experimentally validated targets and biological pathways. By combining publicly available datasets and bioinformatic tools, we aimed to discover common mechanisms through which those unexplored ncRNAs may contribute to inflammation and airway remodeling in obesity-related asthma. The ncRNAs included in this analysis were prioritized based on their reported involvement in obesity and asthma in the literature, and then cross-checked in public databases. The microRNA target genes were obtained from miRWalk, TarBase, and miRTarBase, applying database-specific selection thresholds to ensure reliability. For miRWalk, only experimentally validated interactions with binding energy ≤ −15 kcal/mol, involving at least 15 consecutive base pairs and restricted to the 3′UTR, were included. From TarBase, we retained experimentally verified interactions with a confidence score ≥ 0.6, while from miRTarBase, we considered all experimentally validated entries. For lncRNA, validated targets were identified using DIANA-LncBase v3 and LncTarD v2.0, where all the experimentally validated targets were retrieved. The compiled list of targets was then subjected to functional gene set enrichment analysis using DAVID, with Gene Ontology (GO) terms and signaling pathways identified through KEGG and REACTOME annotations. The enriched pathways were then filtered for statistical significance (FDR < 0.05) and enrichment score (>1.5) according to guidelines by Huang et al. [80]. Among these, pathways of particular interest were selected due to their potential association with known pathophysiological mechanisms of obesity-related asthma. The most prominent enriched pathways were linked to the development of inflammation, airway remodeling, as well as pathways related to metabolic regulation and insulin signaling, which are known to be disrupted in obesity and may contribute to the pathogenesis of asthma in obese individuals. Table 3 summarizes the results of the in silico analyses, highlighting pathways relevant to metabolic processes. Given the large number of target genes, the full raw dataset is not included in the manuscript but is available from the authors upon request. For transparency, the complete enrichment results are summarized in the Supplementary Data (Tables S1 and S2), which include all statistically significant pathways relevant to inflammation, airway remodeling, and metabolic regulation.

The most significantly enriched pathways included those closely related to insulin-like growth signaling, including and IGF1R signaling pathway and SHC-related events triggered by IGF1R. These pathways are most prominent in adipocytes and are involved in cell survival, proliferation, and metabolic homeostasis, suggesting that selected ncRNAs may also influence these processes. Additionally, multiple pathways associated with adipocyte biology were also enriched, such as regulation of lipolysis in adipocytes as well as positive and negative regulation of fat cell differentiation.

The simultaneous enrichment of opposing adipogenic pathways may indicate that different ncRNAs exert various effects on adipogenesis, depending on their targets and the cellular context. An example of such an opposite effect is miR-181a-5p, which targets genes such as *CDS1*, *STK4*, and *AKT1* within the positive regulation of fat cell differentiation, while also inhibiting pro-inflammatory mediators such as *TNF* and *IL6* in the context of negative regulation. A similar pattern was observed for lncRNA H19, which modulates the expression of *AKT1* in adipogenesis-promoting pathways, and also targets *IL6* and *WNT1* in pathways associated with its inhibition. These findings raise the possibility that individual ncRNAs may potentially influence adipogenesis in a bidirectional manner, depending on specific targets and the surrounding signaling environment. Given the established link between dysfunctional adipose tissue and systemic inflammation in obesity-related asthma, clarifying these potential dual roles of ncRNAs represents an important direction for future research.

**Figure 1:**
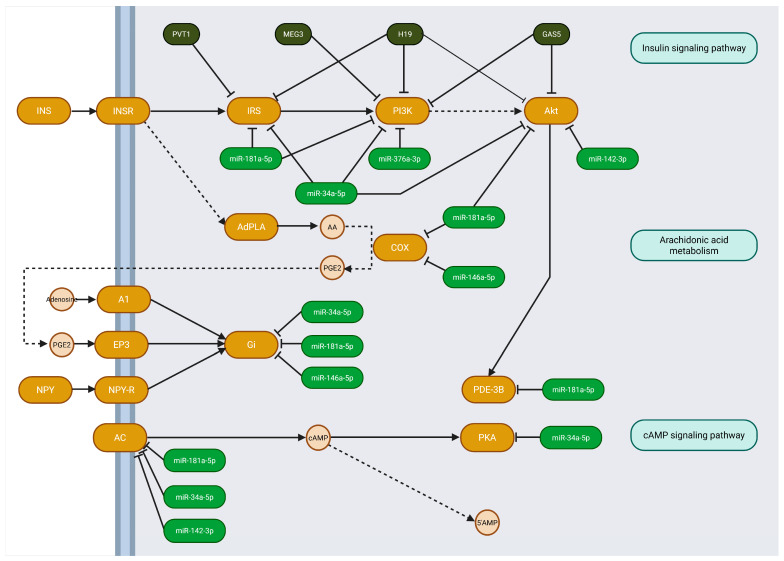
Regulation of lipolysis in adipocytes with annotated ncRNAs interactions and their targets. The *IRS* family includes *IRS1*, *IRS2*, and *IRS4*; the PI3K family includes *PIK3R1*, *PIK3R2*, *PIK3R3*, *PIK3CA*, and *PIK3B*; The *Akt* family involves *AKT1*, *AKT2*, and *AKT3*; the COX family involves *PTGS1* and *PTGS2*; The *Gi* family involves *GNAI1*, *GNAI2*, and *GNAI3*; the *AC* family involves *ADCY1*, *ADCY2*, *ADCY3*, *ADCY4*, *ADCY5*, *ADCY6*, *ADCY7*, *ADCY8*, and *ADCY9*; the *PKA* family involves *PRKACG*, *PRKACA*, and *PRKACB.* Solid arrows indicate direct activation or enzymatic reactions, dashed arrows represent indirect regulation or metabolite transport, and T-shaped lines indicate inhibition.

To further illustrate the potential biological relevance of the identified pathways, we focused on the regulation of lipolysis in adipocytes, due to its functional connection to both obesity and metabolic inflammation. A visual representation of this pathway, annotated with the predicted targets of the selected ncRNAs, is shown in Figure 1. The pathway map was annotated with interactions between ncRNAs and their target genes. Notably, miR-181a-5p and miR-34a-5p emerged as one of the main regulators, targeting multiple components involved in signaling cascades. These include *IRS*, *PI3K*, *PDE3B*, *PKA*, and *Gi* gene families, as well as *AC*, which are part of the insulin and cAMP signaling pathways, along with affecting gene *COX*, encoding a key enzyme in the arachidonic acid metabolism pathway. On the other hand, one of the most prominent lncRNAs in this pathway is H19, which also modulates the expression of *IRS*, *PI3K*, and *Akt* families. Interestingly, based on the results of in silico analysis, we can state that this particular lncRNA also interacts with miR-181a-5p and miR-34a-5p. However, the exact mechanism and its relevance in this specific pathway remain unclear and require further investigation.

Another promising direction for future studies in obesity-associated asthma is the investigation of extracellular vesicles (EVs). EVs are key mediators of intercellular communication, capable of transferring not only proteins, lipids, and cytokines but also regulatory ncRNAs between adipose tissue and pulmonary cells, thereby influencing inflammation and airway remodeling [81,82,83]. Several studies have demonstrated that the ncRNA cargo of EVs reflects disease-specific alterations. For example, 80% of EVs in bronchoalveolar lavage fluid (BALF) originate from airway epithelium, but their composition changes during allergic airway inflammation, with immune cell-derived EVs enriched in pro-inflammatory miRNAs such as miR-223 and miR-142a [84]. Similarly, bronchial epithelial cells exposed to IL-13 release EV-associated miRNAs, including miR-92b, miR-210, and miR-34a, which were validated in nasal lavages of children with severe and non-severe asthma and correlated with lung function [85]. Altered exosomal miRNA signatures have also been observed in bronchoalveolar lavage fluid and serum of patients with asthma, including increased levels of miR-125b [86] and miR-126 [87], as well as miR-185-5p [88] and miR-122-5p [89], some of which discriminated between asthma phenotypes and disease severity. These findings highlight EVs as a potential reservoir of clinically relevant ncRNAs that could serve as minimally invasive biomarkers in blood, sputum, or lavage fluids, and may ultimately contribute to patient stratification and personalized therapies [81,82,83]. However, their functional roles in the pathogenesis of obesity-associated asthma remain largely unexplored, representing an exciting avenue for future research.

## 6. Future Perspectives

Currently, there are no available therapeutic strategies utilizing ncRNAs for the treatment of asthma, obesity, or obesity-related asthma. Ongoing research primarily focuses on identifying potential therapeutic targets and biomarkers, such as lncRNA GAS5 or H19 mentioned above, rather than developing direct interventions. Nevertheless, ncRNAs offer a range of promising therapeutic approaches, including ncRNA inhibitors and synthetic analogs, that hold potential for modulating disease-related pathways. However, several challenges must be addressed before ncRNA-based therapies can be translated into clinical applications. These include ensuring tissue specificity, enhancing stability in circulation by preventing degradation through, e.g., encapsulation in lipid nanocarriers or extracellular vesicles (EVs), and most importantly, guaranteeing patients’ safety by minimizing off-target and side effects.

## Figures and Tables

**Table 1:** Summary of ncRNAs involved in the inflammation in asthma, obesity, and obesity-related asthma.

Disease	miRNA		lncRNA		circRNA		References
	Up	Down	Up	Down	Up	Down	
Asthma	let-7e-5p	miR-135b-5p	AK085865	4930597A21Rik	circDHTKD1	-	miRNA:
	miR-142-3p	miR-149	AK149641	Gm13372	circTXNRD1		[23,28,31,33,34,35,36,37,38,39]
	miR-19a	miR-17	CRNDE	Gm17501			LncRNA:
	miR-223-3p	miR-182-5p	Gm11529	H19			[28,32,36,37,38,40,41,42]
	miR-629-3p	miR-29a-3p	lnc-BAZ2B	PVT1			CircRNA:
		miR-338-3p	MEG3				[31,39]
		miR-451	NKILA				
		miR-892a					
Obesity	miR-122	miR-197-5p	LINK-A	H19	-	circNTRK2	miRNA:
	miR-155	miR-497a-5p	MEG3	PARAIL		circORC5	[24,29,30,43,44,45,46,47,48]
	miR-192	miR-760	SNHG14	TUG1			LncRNA:
	miR-204						[30,45,49,50,51]
	miR-210-3p						CircRNA: [46]
	miR-30e-5p						
	miR-34a						
	miR-34a						
	miR-485-5p						
	miR-378a-3p						
Obesity-related Asthma	miR-26a-1-3p	miR-146a-5p	GAS5	OIP5-AS1	-	-	miRNA:
	miR-376a-3p	miR-181a-5p	HOTAIRM1				[26,52]
		miR-34a-5p	MZF1-AS1				LncRNA:
							[22]

**Table 2:** Summary of ncRNAs involved in airway remodeling in asthma.

Disease	miRNA		lncRNA		circRNA		References
	Up	Down	Up	Down	Up	Down	
Asthma	miR-142-3p	miR-145	PVT1	-	circHIPK3	circ_000002	miRNA
	miR-142-5p	miR-448-5p	MALAT1		circERBB2		
	miR-576-5p	miR-192-5p	TUG1		circ_000259		[64,65,68,69,70,73,75,76,77,78]
		miR-130a-3p	ANRIL				
		miR-451a					lncRNA:
		miR-216a					
		miR-98-5p					[68,69,73,74]
		miR-181b					CircRNA:
		miR-139-5p					[71,72,77,78,79]
		miR-7-5p					

**Table 3:** In silico analysis results—signaling pathways relevant to metabolic processes.

Pathway	Genes	Fold Enrichment	FDR
SHC-related events triggered by IGF1R	NRAS, IGF2, GRB2, KRAS, IGF1, SOS1, HRAS, IGF1R	4.0133	0.0041
Regulation of cellular response to insulin stimulus	NCOA1, NCOA2, BGLAP, CUL3, USO1, KBTBD2, PPARG, ATP2B1	3.7422	0.0337
Positive regulation of fat cell differentiation	CDS1, CEBPB, CREBL2, HTR2A, STK4, MEDAG, ZFP36L1, WIF1, AKT1, LMO1, XBP1, LMO3, ZBTB16, SIRT6, TMEM64, AXIN2, BMP2, SFRP1, CREB1, KLF5, CARM1, ID2, ASXL2, SNAI2, PPARG, WDFY2	2.1718	0.0041
IGF1R signaling cascade	KLB, IRS1, PDE3B, IRS2, PIK3R2, PIK3CB, PIK3R1, FGF1, FGF2, IGF1R, FGF7, NRAS, THEM4, HRAS, PDPK1, IGF2, GAB1, FRS2, PTPN11, IGF1, PIK3CA, GRB2, KRAS, SOS1, FGF10	2.0066	0.0039
Signaling by Type 1 Insulin-like Growth Factor 1 Receptor (IGF1R)	KLB, IRS1, PDE3B, IRS2, PIK3R2, PIK3CB, PIK3R1, FGF1, FGF2, IGF1R, FGF7, NRAS, THEM4, HRAS, PDPK1, IGF2, GAB1, FRS2, PTPN11, IGF1, PIK3CA, GRB2, KRAS, SOS1, FGF10	1.9673	0.0051
Insulin receptor signalling cascade	KLB, IRS1, PDE3B, IRS2, PIK3R2, PIK3CB, PIK3R1, FGF1, FGF2, FGF7, NRAS, GRB10, THEM4, MAPK1, HRAS, MAPK3, PDPK1, GAB1, FRS2, PTPN11, PIK3CA, GRB2, KRAS, SOS1, FGF10	1.9295	0.0069
Negative regulation of fat cell differentiation	YAP1, SMAD2, WWTR1, TGFB1, JAG1, SMAD3, SORT1, WNT3A, ADIPOQ, RORA, SIRT1, TNF, FOXO1, ZFP36L2, VEGFA, BMP2, IL6, DDIT3, ID4, E2F1, ENPP1, WNT1, BMAL1, TRIB2	1.9028	0.0387
Response to glucose	PFKFB2, CDKN1B, ILDR2, IRS2, RASAL2, ELAVL1, THBS1, ACVR1C, SGCB, CASP3, HNF4A, SESN2, GLUL, ZBED3, SREBF1, TCF7L2, EGR1, PRKCB, ADIPOQ, ACVR2B, RPS6KB1, TXNIP, COL6A3, SELENOT, VAMP2, SIDT2	1.9003	0.0274
Fat-cell differentiation	RNASEL, STEAP4, CBY1, FITM2, FOXO1, C1QTNF3, BCL2L13, CCND1, SOX8, TBL1X, WNT1, ZBTB7A, SREBF1, OSBPL8, TCF7L2, EGR2, NEGR1, WNT3A, HMGA2, ARID5B, INHBB, SENP2, KLF4, PIAS1, NR4A2, CLIP3, NR4A3, ID4, CNTN2, PLCB1, ATF5, TRIM32	1.6819	0.0505
Regulation of lipolysis in adipocytes	IRS1, PDE3B, GNAI3, PIK3R3, ADCY2, PIK3R2, IRS2, ADCY1, PIK3CB, PIK3R1, PTGS2, GNAI1, TSHR, ADCY5, GNAI2, ADCY9, PIK3CA, AKT3, GNAS, AKT1, PRKACB, MGLL, PRKG1	1.5495	0.0351

## Data Availability

The original contributions presented in this study are included in the article/Supplementary Materials. Further inquiries can be directed to the corresponding author.

## Supplementary Materials

The following supporting information can be downloaded at: https://www.mdpi.com/article/10.3390/jcm14207161/s1, Table S1: In silico analysis results—signaling pathways relevant to inflammation; Table S2: In silico analysis results—signaling pathways relevant to airway remodeling.
