# Supplementary material for: The Impact of Non-Coding RNA on Inflammation and Airway Remodeling in Asthma Related to Obesity: State-of-the-Art and Therapeutic Perspectives
Source: J Clin Med. 2025 Oct 11;14(20):7161. doi: 10.3390/jcm14207161 (PMC12564001; doi:10.3390/jcm14207161)
Supplement: Supplementary file 1 [file jcm-14-07161-s001.zip › jcm-3865515-supplementary.pdf]

SUPPLEMENTARY MATERIAL

"The impact of non-coding RNA on inflammation and airway remodeling in asthma related to obesity: state-of-art and therapeutic perspectives"

Maria Kachel, Wojciech Langwiński, Aleksandra Szczepankiewicz

Table S1 In silico analysis results – signaling pathways relevant to inflammation.

Pathway	Genes	Fold Enrichment	FDR
Toll Like Receptor 3 (TLR3) Cascade	ATF1, ATF2, UBE2D2, UBE2D3, NLRC5, UBE2D1, HMGB1, PPP2CA, RPS6KA3, MAPK8, CASP8, RPS6KA5, TBK1, IRAK1, IRAK2, TIFA, S100A12, MAPK1, MAP3K8, FADD, BTRC, MAP3K7, IKBKE, MAPK3, SKP1, MAP2K6, MEF2A, JUN, MAP2K1, MEF2C, FBXW11, PPP2R5D, FOS, NFKB1, DUSP6, CREB1, TRAF3, TRAF6, SAA1, IRF7, UBE2N, TAB3, TAB2, TAB1, TP53, BIRC3	1,7416	0,0010
Th17 cell differentiation	RORA, GATA3, AHR, IL1RAP, HIF1A, PPP3CA, PPP3R1, MAPK8, TBX21, IL21R, MAPK1, PLCG1, JAK2, IL12RB1, IL6R, JAK1, MAPK3, SMAD2, JUN, SMAD4, TGFB1, SMAD3, STAT1, STAT3, NFATC3, NFATC2, FOS, FOXP3, TGFBR1, IL2, MTOR, NFKB1, TGFBR2, RUNX1, MAPK13, ZAP70, CD4, IL6, IFNG, IRF4, IL1B, IL2RB, IL6ST, NFKBIE	1,6045	0,0013
Interleukin-6 signaling	SOCS3, IL6, STAT1, STAT3, PTPN11, CBL, IL6ST, JAK2, IL6R, JAK1	3,6484	0,0018
Toll Like Receptor 5 (TLR5) Cascade	ATF1, ATF2, NLRC5, HMGB1, PPP2CA, RPS6KA3, MAPK8, CASP8, RPS6KA5, IRAK1, IRAK2, TIFA, S100A12, MAPK1, MAP3K8, BTRC, MAP3K7, MAPK3, SKP1, MAP2K6, MEF2A, JUN, MAP2K1, MEF2C, MAP3K1, FBXW11, PPP2R5D, FOS, IRAK4, NFKB1, DUSP6, CREB1, TRAF6, PELI1, SAA1, UBE2N, TAB3, TLR10, TAB2, TAB1, TP53, MYD88	1,7377	0,0018
Toll Like Receptor 10 (TLR10) Cascade	ATF1, ATF2, NLRC5, HMGB1, PPP2CA, RPS6KA3, MAPK8, CASP8, RPS6KA5, IRAK1, IRAK2, TIFA, S100A12, MAPK1, MAP3K8, BTRC, MAP3K7, MAPK3, SKP1, MAP2K6, MEF2A, JUN, MAP2K1, MEF2C, MAP3K1, FBXW11, PPP2R5D, FOS, IRAK4, NFKB1, DUSP6, CREB1, TRAF6, PELI1, SAA1, UBE2N, TAB3, TLR10, TAB2, TAB1, TP53, MYD88	1,7377	0,0018
Toll Like Receptor 9 (TLR9) Cascade	ATF1, ATF2, NLRC5, HMGB1, PPP2CA, EEA1, RPS6KA3, MAPK8, CASP8, RPS6KA5, IRAK1, IRAK2, TIFA, S100A12, MAPK1, MAP3K8, BTRC, MAP3K7, MAPK3, SKP1, MAP2K6, MEF2A, JUN, MAP2K1, MEF2C, MAP3K1, FBXW11, PPP2R5D, FOS, IRAK4, NFKB1, DUSP6, CREB1, TRAF6, PELI1, SAA1, IRF7, UBE2N, TAB3, TAB2, IRF5, TLR7, TAB1, TP53, TLR4, MYD88	1,6632	0,0026
Toll Like Receptor 7/8 (TLR7/8) Cascade	ATF1, ATF2, NLRC5, HMGB1, PPP2CA, RPS6KA3, MAPK8, CASP8, RPS6KA5, IRAK1, IRAK2, TIFA, S100A12, MAPK1, MAP3K8, BTRC, MAP3K7, MAPK3, SKP1, MAP2K6, MEF2A, JUN, MAP2K1, MEF2C, MAP3K1, FBXW11, PPP2R5D, FOS, IRAK4, NFKB1, DUSP6, CREB1, TRAF6, PELI1, SAA1, IRF7, UBE2N, TAB3, TAB2, IRF5, TLR7, TAB1, TP53, TLR4, MYD88	1,6722	0,0026
Toll Like Receptor 4 (TLR4) Cascade	ATF1, ATF2, UBE2D2, UBE2D3, ITGB2, UBE2D1, HMGB1, RPS6KA3, RPS6KA5, TBK1, CASP8, MAP3K8, BTRC, IKBKE, MAP3K7, SKP1, MEF2A, MAP2K1, MEF2C, FBXW11, S100A1, PPP2R5D, IRAK3, FOS, IRAK4, DUSP6, CREB1, TRAF3, TRAF6, PELI1, IRF7, TLR4, TP53, TLR2, BIRC3, NLRC5, PPP2CA, MAPK8, IRAK1, IRAK2, TIFA, S100A12, MAPK1, FADD, MAPK3, MAP2K6, JUN, MAP3K1, PTPN11, NFKB1, SAA1, UBE2N, TAB3, TAB2, TAB1, PTPN4, MYD88	1,5562	0,0032
TRAF6 mediated induction of NFkB and MAP kinases upon TLR7/8 or 9 activation	ATF1, ATF2, NLRC5, HMGB1, PPP2CA, RPS6KA3, MAPK8, CASP8, RPS6KA5, IRAK1, IRAK2, TIFA, S100A12, MAPK1, MAP3K8, BTRC, MAP3K7, MAPK3, SKP1, MAP2K6, MEF2A, JUN, MAP2K1, MEF2C, MAP3K1, FBXW11, PPP2R5D, FOS, IRAK4, NFKB1, DUSP6, CREB1, TRAF6, PELI1, SAA1, UBE2N, TAB3, TAB2, TLR7, TAB1, TP53, TLR4, MYD88	1,6754	0,0033
Leukocyte transendothelial migration	ITGB1, ROCK1, ROCK2, PXN, ITGB2, GNAI3, CXCR4, PIK3R3, CD99L2, PIK3R2, PIK3CB, PIK3R1, ARHGAP5, GNAI1, ARHGAP35, ACTG1, GNAI2, ICAM1, RAP1B, CDC42, RAP1A, CTNNA3, PLCG1, RAC1, VAV3, VCAM1, ITGA4, PRKCB, MMP2, CYBB, MSN, PTPN11, MMP9, RHOA, PTK2, MAPK13, AFDN, OCLN, CXCL12, PIK3CA, CLDN12, CTNNB1, VCL, RAPGEF4	1,5077	0,0048
IL-17 signaling pathway	GSK3B, CEBPB, SRSF1, CXCL1, PTGS2, ELAVL1, TNF, CXCL2, HSP90B1, MAPK8, CASP8, TBK1, CASP3, CCL2, MAPK1, FADD, MAP3K7, IKBKE, MAPK4, MAPK3,	1,5481	0,0065

	JUN, IL13, FOS, MMP9, NFKB1, MAPK13, FOSL1, CXCL10, IL6, IFNG, TRAF3, TRAF6, IL1B, FOSB, TAB3, TAB2, ANAPC5		
CD28 dependent PI3K/Akt signaling	CD86, PDPK1, CD80, PIK3R3, PIK3R2, PIK3CB, PIK3R1, MTOR, PIK3CG, PIK3CA, AKT3, AKT1, THEM4, FYN, MAP3K8, RICTOR	2,3782	0,0065
Toll Like Receptor TLR6:TLR2 Cascade	ATF1, ATF2, NLRC5, HMGB1, PPP2CA, RPS6KA3, MAPK8, CASP8, RPS6KA5, IRAK1, IRAK2, TIFA, S100A12, MAPK1, MAP3K8, BTRC, MAP3K7, MAPK3, SKP1, MAP2K6, MEF2A, JUN, MAP2K1, MEF2C, MAP3K1, FBXW11, S100A1, IRAK3, PPP2R5D, FOS, IRAK4, NFKB1, DUSP6, CREB1, TRAF6, PELI1, SAA1, UBE2N, TAB3, TAB2, TAB1, TP53, TLR4, MYD88, TLR2	1,5982	0,0066
Toll Like Receptor TLR1:TLR2 Cascade	ATF1, ATF2, NLRC5, HMGB1, PPP2CA, RPS6KA3, MAPK8, CASP8, RPS6KA5, IRAK1, IRAK2, TIFA, S100A12, MAPK1, MAP3K8, BTRC, MAP3K7, MAPK3, SKP1, MAP2K6, MEF2A, JUN, MAP2K1, MEF2C, MAP3K1, FBXW11, S100A1, IRAK3, PPP2R5D, FOS, IRAK4, NFKB1, DUSP6, CREB1, TRAF6, PELI1, SAA1, UBE2N, TAB3, TAB2, TAB1, TP53, TLR4, MYD88, TLR2	1,5569	0,0109
Toll Like Receptor 2 (TLR2) Cascade	ATF1, ATF2, NLRC5, HMGB1, PPP2CA, RPS6KA3, MAPK8, CASP8, RPS6KA5, IRAK1, IRAK2, TIFA, S100A12, MAPK1, MAP3K8, BTRC, MAP3K7, MAPK3, SKP1, MAP2K6, MEF2A, JUN, MAP2K1, MEF2C, MAP3K1, FBXW11, S100A1, IRAK3, PPP2R5D, FOS, IRAK4, NFKB1, DUSP6, CREB1, TRAF6, PELI1, SAA1, UBE2N, TAB3, TAB2, TAB1, TP53, TLR4, MYD88, TLR2	1,5569	0,0109
Fc epsilon RI signaling pathway	PIK3R3, PIK3R2, PIK3CB, PIK3R1, TNF, NRAS, MAPK8, INPP5D, AKT3, AKT1, MAPK1, FYN, PLCG1, RAC1, HRAS, MAPK3, MAP2K6, VAV3, MAP2K1, PDPK1, IL13, PLA2G4C, PLA2G4A, MAPK13, PIK3CA, GRB2, KRAS, SOS1	1,6130	0,0109
Interleukin-17 signaling	ATF1, ATF2, PPP2CA, RPS6KA3, MAPK8, RPS6KA5, IRAK1, IRAK2, MAPK1, MAP3K8, BTRC, MAP3K7, MAPK3, SKP1, MAP2K6, MEF2A, JUN, MAP2K1, MEF2C, FBXW11, PPP2R5D, FOS, NFKB1, DUSP6, CREB1, TRAF6, UBE2N, TAB3, TAB2, TAB1	1,6957	0,0175
Interferon alpha/beta signaling	IFITM3, IFITM1, RNASEL, IFIT5, IFI35, SAMHD1, IFIT1, IFIT3, OASL, SOCS3, GBP2, JAK1, KPNA1, IFNAR2, PTPN1, EGR1, RSAD2, IFNB1, STAT1, MX2, EIF2AK2, PTPN11, ISG15, IFI27, OAS2, IRF4, IRF1, IRF7, IRF8, IRF5, KPNB1	1,5950	0,0339
Interleukin-10 signaling	IL10, CD86, CCR1, CCL22, CD80, STAT3, LIF, IL18, CXCL1, PTGS2, CXCL2, TNF, ICAM1, IL1A, CXCL10, IL6, IL1B, CCL5, CCL2, TIMP1, JAK1	1,7932	0,0363
Inflammatory bowel disease	RORA, GATA3, TNF, TBX21, IL21R, IL12RB1, IL10, SMAD2, TGFB2, JUN, TGFB1, SMAD3, STAT1, IL13, STAT3, IL18, FOXP3, IL2, NFKB1, IL1A, IL6, IFNG, IL1B, TLR4, TLR2	1,5056	0,0373

Table S2 In silico analysis results – signaling pathways relevant to airway remodeling.

Pathway	Genes	Fold Enrichment	FDR
Adherens junctions interactions	PVR, ACTG1, CDH6, CDH4, CDH2, CDH1, HOXC8, CADM2, ADAM19, TNRC6C, HEYL, AFDN, MOV10, ILF3, ZEB2, AGO3, SP1, AGO4, AGO1, CDH10, AGO2, CDH11, SNAI1, CTNNB1, CDH13, ANGPTL4, TNRC6A, TNRC6B, NECTIN1	2,0418	0,0011
VEGFR2 mediated cell proliferation	PRKCB, PDPK1, SPHK1, PRKCD, ITPR3, NRAS, RASA1, KDR, KRAS, CALM3, PLCG1, CALM1, HRAS, CALM2	2,9571	0,0013
Response of endothelial cells to shear stress	ITGB1, YAP1, CALCRL, ITGB3, ADM, ADCY2, PIK3R2, ADCY1, PIK3CB, ADCY5, PPP2CA, P2RY2, KDR, AKT1, RICTOR, ITGAV, FYN, IKBKE, PRKACB, PTPN1, ANXA2, PDPK1, STAT1, FN1, MTOR, PTK2, NFKB1, MMP14, ADCY9, PIK3CA, GNAQ, GNB2, GNB1, GNB4, GNAS, CTNNB1, PKN2, CALM3, CALM1, CALM2, VCL	1,7693	0,0015
Gap junction	GUCY1B1, TUBAL3, SRC, GNAI3, ADCY2, ITPR3, HTR2A, ADCY1, GNAI1, EGFR, GNAI2, ADCY5, TUBA1C, NRAS, GRM5, MAPK1, DRD1, HRAS, PRKACB, PRKG1, MAPK3, PDGFRB, MAP3K2, GUCY1A2, PDGFRA, MAP2K1, GUCY1A1, PRKCB, TUBB, TUBB2A, ADCY9, GNAQ, CDK1, GNAS, GRB2, KRAS, PLCB1, SOS1	1,6418	0,0019
TGF-beta receptor signaling activates SMADs	ITGB1, USP15, ITGB3, LTBP2, MTMR4, CBL, PPP1CB, XPO1, PMEPA1, ITGB8, ITGAV, SMAD2, TGFB2, SMAD4, TGFB1, SMAD3, TGFBR1, TGFBR2, PPP1CA, SMAD7, TGFBR3, FKBP1A, FKBP1C, STRAP, FBN1	2,0902	0,0021
MAP2K and MAPK activation	MAP2K1, KSR1, YWHAB, SRC, ITGB3, FN1, PEBP1, BRAF, ARRB2, IQGAP1, KSR2, ACTG1, CNKSR2, RAP1B, NRAS, RAP1A, MAPK1, KRAS, HRAS, MARK3, VCL, MAPK3	2,2073	0,0022
MAP kinase activation	ATF1, ATF2, PPP2CA, RPS6KA3, MAPK8, RPS6KA5, IRAK1, IRAK2, MAPK1, MAP3K8, BTRC, MAP3K7, MAPK3, SKP1, MAP2K6, MEF2A, JUN, MAP2K1, MEF2C, FBXW11, PPP2R5D, FOS, NFKB1, DUSP6, CREB1, TRAF6, UBE2N, TAB3, TAB2, TAB1	1,9111	0,0027

Signaling by NOTCH1	HDAC4, NOTCH1, MAML2, HDAC10, HDAC1, ARRB2, RBPJ, HDAC9, HIF1A, DLL1, HDAC6, HDAC7, APH1A, NEURL1B, NCSTN, HEY1, MYC, HEY2, HES1, TBL1X, SKP1, JAG1, FBXW7, ADAM10, MIB1, CDK8, HEYL, KAT2B, ADAM17, NCOR1, NBEA, TBL1XR1, NUMB	1,8142	0,0037
Signalling to ERKs	MAP2K1, RALA, RALB, SHC3, YWHAB, BRAF, FRS2, MAPK13, NRAS, RAP1A, MAPK1, GRB2, KRAS, SOS1, HRAS, RALGDS, CRK, MAPK3	2,1891	0,0087
Notch signaling pathway	NOTCH2, NOTCH3, NUMBL, NOTCH1, CTBP2, MAML2, HDAC1, NOTCH4, DTX3, RBPJ, DLL1, APH1A, LFNG, NCSTN, ATXN1, HEY1, HEY2, ATXN1L, HES1, SPEN, TLE3, JAG1, HEYL, KAT2B, ADAM17, NUMB	1,6669	0,0096
MET activates PTK2 signaling	ITGB1, LAMA5, COL27A1, COL24A1, SRC, ITGA3, HGF, ITGA2, LAMA3, FN1, LAMC2, LAMC1, PTK2, COL1A1, COL2A1, COL5A1, MET	2,2008	0,0109
Cell junction organization	ITGB1, CD151, LAMA3, PXN, TESK1, LAMC2, PVR, ACTG1, CDH6, PARD6B, CDH4, CDH2, CDH1, RSU1, HOXC8, PRKCI, DST, CADM2, ADAM19, TNRC6C, HEYL, AFDN, MOV10, ILF3, ZEB2, AGO3, SP1, AGO4, AGO1, CDH10, KRT14, AGO2, CLDN12, CDH11, SNAI1, CTNNB1, CDH13, ITGA6, ANGPTL4, SDK2, TNRC6A, TNRC6B, PALS1, LIMS1, NECTIN1	1,5569	0,0109
Signaling by TGF-beta Receptor Complex in Cancer	SMAD2, FKBP1A, SMAD4, TGFB1, SMAD3, FKBP1C, TGFBR1, TGFBR2	3,5673	0,0111
MAPK3 (ERK1) activation	IL6, MAP2K1, CDK1, PTPN11, JAK2, IL6R, JAK1, MAPK3	3,5673	0,0111
MAPK6/MAPK4 signaling	PSMD12, PSMD11, HSPB1, FOXO3, KALRN, FOXO1, CDC14B, CDC42, DNAJB1, CCND3, XPO1, PSMB5, MYC, IGF2BP1, RAC1, PAK2, PRKACB, MAPK4, JUN, NCOA3, SEPTIN7, TNRC6C, MOV10, AGO3, AGO4, AGO1, PSMC2, AGO2, CDK1, MAPKAPK5, TNRC6A, TNRC6B	1,6679	0,0167
RAF-independent MAPK1/3 activation	DUSP5, MAP2K1, DUSP1, PTPN11, DUSP6, IL6, PEA15, CDK1, MAPK1, JAK2, IL6R, JAK1, MAPK3	2,3715	0,0211
Negative regulation of MAPK pathway	DUSP5, MAP2K1, KSR1, YWHAB, DUSP1, PEBP1, PPP2R5A, BRAF, PPP2R5D, PPP2R5C, DUSP6, PPP2CA, NRAS, PPP2R5E, MAPK1, KRAS, HRAS, MARK3, PTPN3, MAPK3	1,8666	0,0293
Cell-cell junction organization	PVR, ACTG1, CDH6, PARD6B, CDH4, CDH2, CDH1, HOXC8, PRKCI, CADM2, ADAM19, TNRC6C, HEYL, AFDN, MOV10, ILF3, ZEB2, AGO3, SP1, AGO4, AGO1, CDH10, AGO2, CLDN12, CDH11, SNAI1, CTNNB1, CDH13, ANGPTL4, SDK2, TNRC6A, TNRC6B, PALS1, NECTIN1	1,5332	0,0420
